# Establishing a research agenda for early-onset colorectal cancer

**DOI:** 10.1371/journal.pmed.1002577

**Published:** 2018-06-01

**Authors:** Caitlin C. Murphy, Amit G. Singal

**Affiliations:** 1 Department of Clinical Sciences, University of Texas Southwestern Medical Center, Dallas, Texas, United States of America; 2 Department of Internal Medicine, University of Texas Southwestern Medical Center, Dallas, Texas, United States of America

## Abstract

In this month's Editorial Caitlin C. Murphy and Amit Singal discuss the increasing incidence of early-onset colorectal cancer in patients under the age of 50.

## Background

Colorectal cancer (CRC) incidence and mortality trends have evolved strikingly in recent decades [1,2]. Since the late 1980s, incidence and mortality have declined among older adults (age ≥50 years) in the United States, with particularly dramatic declines in those over the age of 65 years. These improvements are likely due to a combination of screening [3,4], changes in the prevalence of certain CRC risk and protective factors (e.g., smoking, aspirin use) [5,6], and advances in treatment [7]. CRC screening with colonoscopy, sigmoidoscopy, or stool-bases tests facilitates early detection and removal of premalignant lesions (i.e., adenomatous polyps) [8] and is recommended for average-risk persons beginning at age 50 years [9].

In contrast to declines in older populations, the incidence of invasive CRC is increasing in younger adults [10,11]. Starting around 1990, incidence increased in this age group (20–49 years) from 8.6 per 100,000 to 12.5 per 100,000 [12]. The largest absolute increases in incidence have occurred among 40–49-year-olds, from 18.2 per 100,000 in 1992 to 26.5 per 100,000 in 2015. Mortality rates have increased slightly during the same period, ranging from 5.4 to 6.5 per 100,000 among the 40–49-year age group. Similar increases have been reported across the globe [1,13]—from France [14] to Canada [15] to Australia [16,17]. These trends have received research and media attention, with intense speculation for why incidence has increased. As a first step, we outline important considerations for establishing a research agenda to advance our understanding of etiological and diagnostic factors contributing to early-onset CRC.

## Increases in early-onset CRC have been primarily driven by higher rates of rectal cancer

Early-onset CRC has not increased uniformly by anatomic sublocation. While rectal cancer incidence in younger adults increased from 3.0 per 100,000 in 1992 to 4.7 per 100,000 in 2015, there were smaller increases in proximal colon cancer during this period [12]. Differences in incidence and distribution of early-onset CRC by sublocation underscore the importance of teasing apart risk factors for colon versus rectal cancer. Generally, risk factors fall into one of three categories: 1) decreased risk of colon cancer but no association with rectal cancer (e.g., dietary intake of folate and calcium); 2) increased risk of colon cancer but no association with rectal cancer (e.g., processed meat, family history); and 3) increased risk of both colon and rectal cancer (e.g., smoking, obesity) [18]. Future etiological studies of early-onset CRC should examine risks separately—or how associations may be modified—by sublocation.

## Efforts to understand early-onset CRC must account for increasing use of colonoscopy in younger adults

Early-onset CRC may reflect earlier detection via colonoscopy, prompting discussion on the contribution of diagnostic factors (e.g., screening, case ascertainment, practice patterns) to the observed incidence trends. Increasing incidence with stable mortality rates are often seen when greater use of screening or diagnostic technology detects cancer earlier. Although incidence of early-onset CRC has increased dramatically, corresponding mortality rates have increased only slightly, and in the youngest age groups, mortality has remained stable [12]. Colonoscopy in younger adults increased by 30% from 2001 to 2009, which parallels increases in incidence during the same period [19]. Although it is not clear whether colonoscopy at younger ages represents overuse, symptomatic assessment, or high-risk screening (e.g., for family history), this increase occurred consistently across age groups, sex, and geographic region.

## Germline mutations explain only a minority of early-onset CRC cases

Only 15%–20% of patients with early-onset CRC have a germline mutation (e.g., APC, MLH1, MUTHY, STK11, SMAD4, TP53), and of these, about half have Lynch Syndrome [20]. Although the prevalence of hereditary syndromes is higher among patients with early-onset CRC compared to the overall CRC patient population, it raises questions concerning the other 80%–85% without a germline mutation but who may be at increased risk of CRC due to family history or other unknown susceptibility genes. Nearly half of patients with early-onset CRC do not have a known family history of CRC [21], but this may be underreported given limited approaches to systematically collect family history in primary care. Alternatively, the minority of cases attributed to germline mutations and/or familial risk may underscore the role of environmental and lifestyle-related factors involved in early-onset disease.

## Persistent racial disparities in early-onset CRC may provide additional insight

CRC disproportionately affects blacks—higher incidence rates and smaller declines in incidence among the screen-eligible population [3]. Although whites have experienced a larger relative increase in incidence of early-onset CRC, absolute incidence rates remain higher among young blacks (14.6 versus 12.4 per 100,000 among blacks and whites, respectively) [12]. Because blacks have higher incidence of early-onset CRC than whites and the age-related acceleration in incidence starts at a younger age, some professional organizations recommend initiating CRC screening at an earlier age, such as 45 years [22]. Racial differences in the distribution of CRC risk factors, such as earlier onset of type 2 diabetes or childhood obesity, may explain some of these disparities and provide additional insight into mechanisms of early-onset CRC.

## Birth cohort effects point to exposures during early childhood and adolescence that may increase CRC risk

Early-onset CRC has increased across successive birth cohorts, particularly for those born after 1950 [10]. Birth cohort effects point to early life exposures—or exposures accumulated throughout the life course—that may increase risk of cancer. For example, younger age at smoking initiation and longer duration of smoking increases risk of lung cancer. Reconsidering the timing and duration of well-established CRC risk factors, such as obesity, may help to identify windows of exposure most susceptible to risk. A growing body of evidence suggests birth weight [23] and childhood obesity [24] increase risk of CRC in adulthood. Others have suggested antibiotic use during infancy or childhood, which increased dramatically during the 1980s [25], may influence microbial diversity [26] and increase risk of cancer in adults [27–29].

## Conclusion

In summary, CRC incidence and mortality rates have declined among older adults, in part because of increased screening uptake among persons over the age of 50 years. These declines have not occurred in younger adults, where incidence has increased. Increasing incidence of early-onset CRC has led to enthusiasm for lowering the recommended age to initiate CRC screening. There are clear benefits to screening in older populations [3], and the temptation is to believe that benefits would be similar for younger adults. However, in an era of precision cancer screening [30], recommendations of when to initiate CRC screening should consider individual differences in risk and expected benefits expressed in absolute terms—not solely on temporal trends in incidence [31]. Potential harms, such as cost and colonic perforation or other adverse events [32], should also be considered. Studying population shifts in the distribution of CRC risk factors, as well as diagnostic practices, may advance our understanding of early-onset CRC and its causes. These data are also important to guide precision screening efforts, improve treatment strategies, and identify subgroups (defined by age and risk level) who may be at the highest risk of CRC.

## Abbreviation

CRC: Colorectal cancer
